# Trends in adolescent COVID-19 vaccination receipt and parental intent to vaccinate their adolescent children, United States, July to October, 2021

**DOI:** 10.1080/07853890.2022.2045034

**Published:** 2022-03-03

**Authors:** Kimberly H. Nguyen, Kimchi Nguyen, Megan Geddes, Jennifer D. Allen, Laura Corlin

**Affiliations:** a Department of Public Health and Community Medicine, Tufts University School of Medicine, Boston, MA, USA; b Department of Medicine, Children’s Hospital, Boston, MA, USA; c Department of Community Health, Tufts University, Medford, MA, USA; d Department of Civil and Environmental Engineering, Tufts University School of Engineering, Medford, MA, USA

**Keywords:** COVID-19 vaccine, vaccine hesitancy, vaccine confidence, disparities, adolescents

## Abstract

**Introduction:**

There was a five-fold increase in COVID-19 hospitalization case counts among children and adolescents between June and October 2021. However, polls suggest that adolescent COVID-19 vaccination coverage has plateaued in the United States.

**Methods:**

Using the Census Bureau’s Household Pulse Survey, we assessed trends in COVID-19 vaccination among adolescents ages 12–17 years, parents’ intention to vaccinate their adolescent children, and their reasons for not intending to vaccinate their children from July to October 2021 using a large, nationally representative survey of U.S. households (*n* = 59,424). Trends in COVID-19 adolescent vaccination coverage, nationally and by sociodemographic characteristics, factors associated with adolescent vaccination status and parental intent to vaccinate their adolescent children, as well as changes in reasons for non-vaccination were examined using regression models.

**Results:**

Receipt of ≥1 dose of a COVID-19 vaccine among adolescents ages 12–17 years increased five percentage points, from 56% (July) to 61% (October), with significant increases across most sociodemographic variables. However, there were no significant changes in parental intention to vaccinate their adolescent children during the same time period. Approximately one-quarter of parents were unsure about or reluctant to vaccinate their children, which remained consistent from July to October. Among those who had not vaccinated their children, lack of trust in the government and vaccines, and the belief that the COVID-19 vaccine is not needed or effective, was higher in October compared to July.

**Conclusions:**

Parental intention to vaccinate their children has remained relatively stable throughout the late summer and early fall of 2021. Encouraging paediatricians to discuss the importance and safety of COVID-19 vaccines, addressing concerns and misinformation, as well as recommending and offering vaccines are important for increasing parental confidence in vaccines as well as vaccination uptake among adolescents.

**KEY MESSAGE**
- Receipt of ≥1 dose of a COVID-19 vaccine among adolescents ages 12–17 years increased five percentage points, from 56% (July) to 61% (October), with significant increases across most sociodemographic variables.
- Approximately one quarter of parents were unsure about or reluctant to vaccinate their children, which remained consistent from July to October.
- Encouraging paediatricians to discuss the importance and safety of COVID-19 vaccines, addressing concerns and misinformation, as well as recommending and offering vaccines is important for increasing parental confidence in vaccines as well as vaccination uptake among adolescents.

## Introduction

The first COVID-19 vaccines became available in the U.S. under an Emergency Use Authorization for individuals age 16 years and older in December 2020, and for children ages 12–15 years in May 2021 [1,2]. Whereas COVID-19 vaccination coverage (≥1 dose) among adults was 80% in September 2021, a poll found that less than half of parents (48%) of vaccine-eligible children ages 12–17 years reported that their child had received at least one dose of a COVID-19 vaccine during the same time period [3,4]. In addition, approximately one in five parents (21%) reported that they would “definitely not” get a vaccine for their adolescent child [4]. Data suggests that adolescent vaccination coverage is lagging in some parts of the U.S., although exact reasons for the plateau are not known [5].

From June to August 2021, hospitalization case counts among children and adolescents rose nearly five-fold, coinciding with the increased spread of the highly transmissible SARS-CoV-2 Delta variant, increased social gatherings and travel, and a relaxation in social distancing and other preventive measures [6–8]. Furthermore, the re-opening of schools for in-person learning, and resuming of sports and other extracurricular activities, amplifies the need for high COVID-19 vaccination coverage among age-eligible children.

Previous studies have included small, non-probability samples, or did not examine changes in adolescent COVID-19 vaccination coverage during the summer and fall months of 2021. Furthermore, parental intent to vaccinate adolescent children, or reasons for not vaccinating, may have changed over time since the vaccines were first approved for adolescents [9]. Assessing trends in vaccination coverage from July to October is important to monitor as cases among children peaked during the summer of 2021 due to the highly transmissible Delta variant, as well as to assess vaccination coverage once in-person learning resumed throughout most of the country. This study assessed trends in COVID-19 vaccination coverage among adolescent children ages 12–17 years from July to October 2021, and changes in parents’ intent to vaccinate their adolescent children and their reasons for non-vaccination using a large, nationally representative survey of U.S. households. Understanding disparities in adolescent vaccination coverage, as well as reasons for not vaccinating, are important for developing appropriate interventions and communication strategies to increase uptake and confidence in COVID-19 vaccines among this population.

## Methods

To assess trends in and factors associated with COVID-19 vaccination coverage (≥1 dose) among children ages 12–17 years (hereafter referred to as adolescents), data from five waves (21 July to 11 October 2021) of the Household Pulse Survey were analyzed [10]. The survey design of the HPS has been described previously [11,12]. The response rates for five waves of data collection (July 21 to August 2, August 4–16, August 18–30, September 1–13, September 15–27, and September 29 to October 11) ranged from 5.6 to 6.1% [12]. Sample sizes of respondents (hereafter referred to as “parents”) with children ages 12–17 years living in the household for each data collection cycle ranged from 8,522 to 10,817 for a total of 59,424 participants. The Tufts University Health Sciences Institutional Review Board reviewed the study and concluded that this secondary analysis based on freely available data does not involve human participants. IRB number: STUDY00001925. The original survey collected data using telephone interviews, and participation in the interview constituted consent. [13].

Among households with children ages 12–17 years, respondents were asked: “Have any of the children aged 12–17 years living in your household received at least one dose of a COVID-19 vaccine?” [yes/no/don’t know]. Among those who answered “no,” respondents were asked about their intent to vaccinate children: “Now that vaccines to prevent COVID-19 are available to most children between ages 12 and 17, will the parents or guardians of children ages 12–17 living in your household…” Response options were definitely, probably, be unsure about, probably not, or definitely not get the children a vaccine, or “I do not know the plans for vaccination of children aged 12–17 living in my household.” Because measuring intent over time would show bias as more people get vaccinated (reducing the sample size of those who are asked about intent), parental intent was defined as having a child who had been vaccinated or the parent was “definitely” or “probably” likely to get them vaccinated. Among respondents who did not report that they had already vaccinated their child or did not definitely plan to get their child vaccinated or did not know the vaccination plans for children, respondents were asked reasons for not getting vaccinated.

Reasons for not getting vaccinated were assessed by the following question: “Which of the following, if any, are reasons that the parents or guardians of children ages 12–17 living in your household [only probably will/probably won’t/definitely won’t/are unsure about whether to] get a COVID-19 vaccine for the children?” Response options, in which respondents could select all that apply, were: 1) Concern about possible side effects of a COVID-19 vaccine for children; 2) Plan to wait and see if it is safe and may get it later, 3) Not sure if a COVID-19 vaccine will work for children, 4) Don’t believe children need a COVID-19 vaccine, 5) The children in this household are not members of a high-risk group, 6) The children’s doctor has not recommended it, 7) Other people need it more than the children in this household do right now, 8) Concern about missing work to have the children vaccinated, 9) Unable to get a COVID-19 vaccine for children in this household, 10) Parents or guardians in this household do not vaccinate their children, 11) Don’t trust COVID-19 vaccines, 12) Don’t trust the government, 13) Concern about the cost of a COVID-19 vaccine, and 14) Other.

Sociodemographic factors assessed were respondent age group [18–49, 50–64, ≥65 years], respondent sex, respondent race/ethnicity [Hispanic, non-Hispanic (NH) Asian, non-Hispanic Black, non-Hispanic white, non-Hispanic other/multiracial], respondent educational attainment [high school equivalent or less, some college or Bachelor’s degree, higher than Bachelor’s degree], annual household income [<$35000, $35000–49999, $50000–74999, ≥$75000, did not report], respondent health insurance status [covered, not], respondent COVID-19 vaccination status [vaccinated, not], region [Northeast, Midwest, West, South^1^],school type of adolescent for households with only children aged 12–17 [only public school, only private school, only homeschool, combination of school types, no enrollment], and number of children aged 12–17 in the household among households with only children in this age range.

Trends in vaccination coverage and intent were assessed for each survey wave and by sociodemographic characteristics through multivariable regression. Factors associated with and differences in adolescent vaccination coverage and parental intent to vaccinate their adolescent children from July to October were examined using regression models for the difference in proportion between the earliest and latest survey waves. Proportions and differences in reasons for not getting vaccinated were assessed for the months of July and October. Analyses accounted for the survey design and weights to ensure a representative sample in SAS (version 9.4; SAS Institute Inc.) and Stata (version 16.1).

## Results

**Figure 1.**
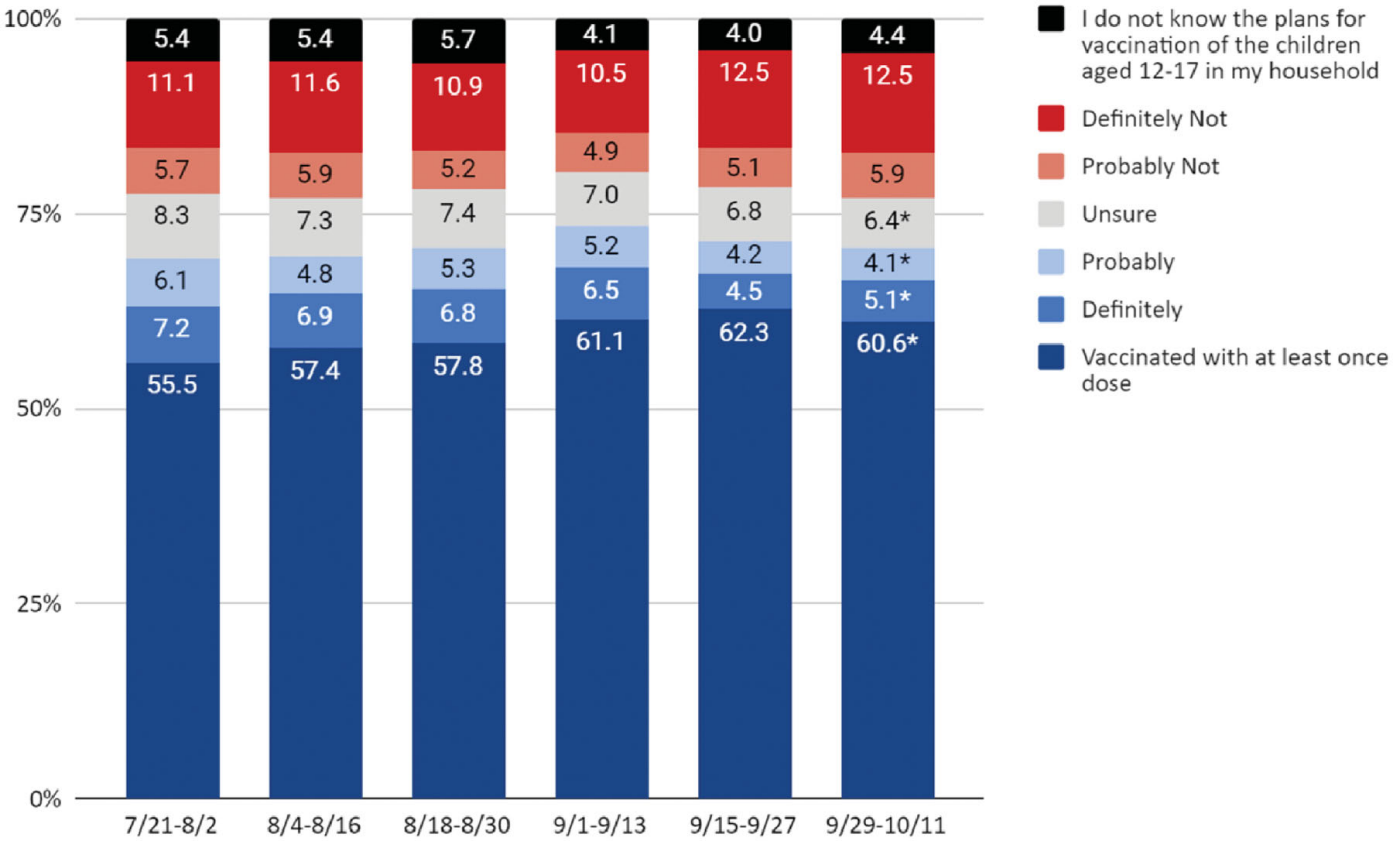
Adolescent COVID-19 vaccination status and parental intent to vaccinate adolescent children from 21 July 2021 to 11 October 2021, United States, Household Pulse Survey. *Statistically significant using linear regression to estimate the difference in proportions between first period of data collection and the latest period of data collection.

From 29 September to 11 October 2021, approximately 61% of adolescents had received ≥1 dose of a COVID-19 vaccine, 9% of adults would definitely or probably get the vaccine for their adolescent child, 6% were unsure, 18% would definitely not or probably not get a vaccine, and 4% do not know the vaccination plans of children in the household (Figure 1). Nationally, there was a five percentage point increase (from 56 to 61%) in receipt of ≥1 dose of a COVID-19 vaccine among adolescents ages 12–17 years from July to October, 2021 (Table 1 and Figure 1). However, there was a decrease in percentage of parents who definitely will (prevalence difference [PD] = −2.1, 95%CI: −3.8, −0.4), probably will (PD = −2.0, 95%CI: −3.4, −0.6), and were unsure about vaccinating their children (PD = −1.9, 95%CI: −3.2, −0.6) Figure 1). There were no changes in percentage of parents who definitely will not or probably will not get the vaccine for their adolescent children, which ranged from 17% in July to 18% in October (Figure 1).

**Table 1.** Trends in COVID-19 vaccination status (≥1 dose of COVID-19 vaccine) among adolescents ages 12–17 years, by respondent sociodemographic characteristics and survey week, United States, Household Pulse Survey, 21 July 2021 to 11 October 2021.

	7/21–8/2		8/4–8/16		8/18–8/30		9/1–9/13		9/15–9/27		9/29–10/11		Difference		9/29–10/11	
	*n* = 10,212		*n* = 10,817		*n* = 10,796		*n* = 9,862		*n* = 9,215		*n* = 8,522				*n* = 3,799	
	%	(95% CI)	%	(95% CI)	%	(95% CI)	%	(95% CI)	%	(95% CI)	%	(95% CI)	%	(95%CI)	aPR^a^	(95%CI)
**All Adolescents (12–17 years)**	55.5	(53.8, 57.1)	57.4	(55.2, 59.6)	57.8	(55.9, 59.7)	61.1	(59.2, 62.9)	62.3	(60.8, 63.9)	60.6	(58.2, 62.9)*	5.1	(2.2, 8.0)		
**Characteristics of Respondent**																
** Age Group (years)**																
** **18–49	52.8	(50.8, 54.8)	54.4	(52.0, 56.8)	55.2	(52.9, 57.6)	59.0	(59.2, 62.0)	58.3	(56.2, 60.4)	57.6	(54.8, 60.4) *	4.8	(1.4, 8.2)	1	referent
** **50–64	66.0	(62.9, 69.1)	66.6	(62.3, 70.9)	65.9	(62.0, 69.8)	67.7	(63.7, 71.6)	74.4	(71.7, 77.1)	69.3	(64.5, 74.0) *	3.3	(−2.4, 9.0)	0.97	(0.91, 1.04)
** **≥65	46.1	(38.7, 53.4)	60.7	(52.5, 68.9)	57.8	(48.4, 67.2)	58.6	(49.9, 67.3)	61.3	(53.2, 69.4)	61.7	(53.5, 70.0)	15.6	(4.6, 26.6)	0.87	(0.74, 1.03)
** Sex**																
** **Male	56.8	(53.6, 59.9)	58.0	(54.3, 61.8)	58.9	(55.7, 62.0)	63.4	(60.3, 66.5)	63.7	(60.7, 66.8)	61.4	(57.7, 65.1)*	4.6	(−0.3, 9.5)	1	referent
** **Female	54.3	(52.0, 56.6)	56.8	(54.6, 59.1)	57.0	(54.8, 59.1)	59.1	(56.7, 61.5)	61.1	(59.4, 62.9)	59.9	(57.4, 62.4)*	5.6	(2.2, 9.0)	0.92	(0.86, 0.97)
** Race/ethnicity**																
** **Non-Hispanic white	55.3	(53.0, 57.6)	54.1	(51.9, 56.2)	55.6	(53.7, 57.6)	60.0	(57.8, 62.2)	60.1	(58.2, 62.0)	59.3	(56.4, 62.2)*	4.0	(0.3, 7.7)	1	referent
** **Non-Hispanic black	44.1	(39.1, 49.1)	47.2	(40.3, 54.0)	51.4	(44.7, 58.1)	53.7	(48.5, 58.9)	60.3	(54.9, 65.6)	47.7	(40.7, 54.6)	3.6	(−5.0, 12.2)	0.95	(0.82, 1.11)
** **Non-Hispanic Asian	84.8	(80.0, 89.6)	64.8	(60.5, 69.1)	84.6	(79.1, 90.1)	85.7	(78.7, 92.8)	86.1	(78.5, 93.7)	89.5	(85.3, 93.8)	4.7	(−1.7, 11.1)	1.11	(1.05, 1.17)
** **Non-Hispanic other/multiple races	55.2	(47.4, 62.9)	85.9	(80.9, 90.8)	44.2	(36.2, 52.2)	44.2	(35.8, 52.6)	59.3	(51.3, 67.3)	53.9	(47.2, 60.7)	−1.3	(−11.6, 9.0)	0.95	(0.80, 1.13)
** **Hispanic	55.7	(51.1, 60.4)	48.6	(38.2, 59.1)	63.0	(58.4, 67.5)	64.1	(59.0, 69.2)	62.8	(58.2, 67.5)	63.7	(58.1, 69.4)*	8.0	(0.7, 15.3)	1.10	(1.01, 1.21)
** Educational Status**																
** **High school or less	47.9	(44.6, 51.2)	48.4	(44.2, 52.6)	49.2	(45.7, 52.7)	53.7	(49.5, 57.9)	55.9	(52.8, 59.0)	52.3	(48.0, 56.6) *	4.4	(−1.0, 9.8)	1	referent
** **Some college or college graduate	57.9	(55.7, 60.2)	61.7	(60.0, 63.4)	60.7	(58.4, 63.1)	63.6	(62.2, 65.0)	63.3	(61.5, 65.1)	63.2	(60.8, 65.7) *	5.3	(2.0, 8.6)	1.07	(0.98, 1.17)
** **Above college graduate	74.2	(71.9, 76.6)	74.4	(71.5, 77.4)	79.0	(76.3, 81.8)	79.0	(76.2, 81.8)	79.9	(77.3, 82.6)	80.4	(77.7, 83.1) *	6.2	(2.6, 9.8)	1.15	(1.05, 1.25)
** Annual Household Income**																
** **Less than $35,000	42.4	(37.2, 47.7)	42.0	(36.7, 47.3)	45.7	(40.9, 50.4)	55.9	(50.6, 61.2)	57.3	(52.3, 62.3)	55.9	(50.8, 61.1) *	13.5	(6.1, 20.9)	1	referent
** **$35,000 – $49,999	53.7	(46.2, 61.2)	59.0	(50.9, 67.1)	55.4	(49.0, 61.8)	54.9	(47.7, 62.0)	55.1	(49.7, 60.6)	59.7	(53.3, 66.1)	6.0	(−3.9, 15.9)	0.95	(0.82, 1.09)
** **$50,000 – $74,999	54.2	(48.7, 59.8)	59.8	(54.6, 65.0)	60.2	(55.4, 64.9)	56.1	(50.0, 62.1)	59.0	(52.7, 64.3)	62.3	(57.1, 67.6)	8.1	(0.5, 15.7)	0.88	(0.76, 1.02)
** **$75,000 and above	66.5	(64.0, 69.0)	67.9	(65.1, 70.6)	69.4	(67.1, 71.1)	72.7	(70.3, 75.2)	71.9	(69.6, 74.3)	72.4	(62.8, 67.6) *	5.9	(2.3, 9.5)	0.96	(0.88, 1.05)
** **Did not report	52.6	(49.1, 56.2)	53.9	(50.0, 57.7)	52.2	(48.3, 56.2)	55.4	(51.4, 59.4)	58.1	(54.3, 62.0)	51.7	(47.4, 56.1)	−0.9	(−6.5, 4.7)	0.85	(0.76, 0.95)
** Insurance Status**																
** **Covered by some type of health insurance	58.1	(56.1, 60.2)	59.4	(56.8, 62.0)	61.7	(59.8, 63.6)	65.2	(63.2, 67.2)	65.4	(63.8, 67.0)	65.2	(62.8, 67.6) *	7.1	(3.9, 10.3)	1	referent
** **Not covered by any type of health insurance	45.0	(37.0, 53.0)	52.0	(44.7, 59.3)	40.4	(33.4, 47.4)	51.5	(44.6, 58.4)	56.3	(48.9, 63.7)	54.4	(44.1, 64.7) *	9.4	(−3.6, 22.4)	1.03	(0.88, 1.20)
** COVID-19 Status**																
** **Previously diagnosed with COVID-19	46.2	(41.9, 50.5)	44.7	(40.5, 48.8)	51.5	(47.2, 55.8)	52.6	(47.6, 57.6)	52.6	(47.8, 57.3)	51.6	(46.5, 56.8)	5.4	(−1.3, 12.1)	1	referent
** **Not previously diagnosed with COVID-19	58.4	(56.6, 60.3)	61.1	(58.6, 63.6)	60.4	(58.4, 62.4)	64.7	(62.5, 66.8)	65.9	(64.0, 67.8)	65.0	(62.8, 67.3)	6.6	(3.7, 9.5)	1.07	(0.99, 1.16)
** COVID-19 Vaccination Status (≥1 dose)**																
** **No	9.2	(6.2, 12.3)	8.1	(5.1, 11.0)	10.0	(7.5, 12.4)	9.8	(7.8, 12.2)	9.0	(6.7, 11.2)	9.1	(7.0, 11.3)	−0.1	(−3.8, 3.6)	1	referent
** **Yes	70.6	(68.9, 72.4)	73.6	(71.4, 75.7)	73.5	(71.3, 75.7)	76.7	(75.1, 78.3)	78.8	(77.2, 80.4)	76.9	(74.5, 79.4)*	6.3	(3.3, 9.3)	5.67	(3.78, 8.51)
** Region** ^b^																
** **Northeast	58.7	(53.2, 64.2)	62.4	(56.3, 68.5)	65.4	(60.6, 70.2)	68.9	(63.1, 74.7)	69.3	(64.2, 74.4)	64.2	(58.5, 69.9)*	5.5	(−2.4, 13.4)	1	referent
** **Midwest	53.8	(49.7, 57.9)	57.6	(53.5, 61.7)	57.3	(53.1, 61.5)	58.9	(54.8, 63.1)	58.7	(55.1, 62.4)	56.7	(52.1, 61.4)	2.9	(−3.3, 9.1)	0.99	(0.92, 1.06)
** **South	50.5	(47.7, 53.3)	49.2	(46.0, 52.3)	51.4	(48.5, 54.3)	55.8	(52.7, 58.9)	58.2	(54.9, 61.4)	56.0	(52.4, 59.6)*	5.5	(0.9, 10.1)	0.94	(0.86, 1.03)
** **West	62.5	(58.7, 66.4)	67.8	(64.7, 70.9)	63.9	(60.0, 67.8)	66.0	(61.8, 70.3)	67.4	(63.9, 70.8)	68.4	(64.9, 71.9)*	5.9	(0.7, 11.1)	1.02	(0.95, 1.08)
** School Type of adolescent (assessed for households with only children ages 12–17)**																
** **Only public school	64.8	(61.5, 68.1)	64.9	(62.1, 67.6)	65.3	(62.2, 68.4)	69.8	(67.3, 72.3)	71.0	(67.9, 74.0)	68.5	(65.5, 71.6)*	3.7	(−0.8, 8.2)	1	referent
** **Only private school	71.2	(61.7, 80.8)	69.1	(61.3, 77.0)	69.2	(61.2, 77.2)	65.8	(54.8, 76.9)	74.2	(66.4, 82.0)	81.9	(76.1, 87.8)*	10.7	(−0.5, 21.9)	1.03	(0.96, 1.11)
** **Only homeschool	58.0	(45.6, 70.4)	41.9	(29.6, 54.2)	43.2	(33.0, 53.4)	50.7	(38.4, 63.0)	41.2	(27.8, 54.6)	42.4	(29.6, 55.1)	−15.6	(−33.4, 2.2)	0.79	(0.57, 1.08)
** **Other combination of schools	58.2	(45.7, 70.6)	54.0	(38.8, 69.2)	70.7	(58.4, 83.0)	54.3	(34.8, 73.7)	60.0	(44.1, 75.9)	68.9	(52.6, 85.1)	10.7	(−9.8, 31.2)	1.02	(0.92, 1.14)
** **None	60.4	(50.7, 70.1)	46.8	(34.8, 58.8)	54.5	(41.6, 67.3)	52.9	(38.1, 67.7)	62.8	(51.7, 74.0)	65.7	(52.7, 78.6)	5.3	(−10.9, 21.5)	1.09	(0.89, 1.33)
** Number of children ages 12–17 (in households with only children ages 12–17)**																
** **1	62.8	(59.7, 66.0)	64.4	(61.5, 67.4)	64.5	(61.9, 67.1)	67.1	(64.0, 70.1)	69.6	(67.6, 71.7)	66.8	(63.4, 70.1)*	4.0	(−0.6, 8.6)	1	referent
** **2	65.2	(60.3, 70.1)	63.1	(58.3, 67.9)	65.0	(60.7, 69.4)	65.6	(60.7, 70.4)	67.1	(62.0, 72.3)	68.7	(64.1, 73.2)*	3.5	(−3.2, 10.2)	1.00	(0.92, 1.08)
** **≥3	56.4	(47.4, 65.4)	44.8	(35.4, 54.3)	50.9	(41.1, 60.6)	60.6	(49.0, 72.1)	55.8	(46.0, 65.7)	58.7	(47.0, 69.5)	2.3	(−11.8, 16.4)	1.03	(0.91, 1.16)

aPR: adjusted prevalence ratio; CI: confidence interval.

*p-value for trend from July to October significant at <.001.

^a^Prevalence ratio adjusted for age, sex, race/ethnicity, educational status, annual household income, insurance status, previous COVID-19 diagnosis, region, school type of adolescent, and number of children in household.

^b^Regions are defined as the following: Northeast – Connecticut, Maine, Massachusetts, New Hampshire, Rhode Island, Vermont, New Jersey, New York, Pennsylvania; Midwest – Indiana, Illinois, Michigan, Ohio, Wisconsin, Iowa, Kansas, Minnesota, Missouri, Nebraska, North Dakota, South Dakota; South – Delaware, District of Columbia, Florida, Georgia, Maryland, North Carolina, South Carolina, Virginia, West Virginia, Alabama, Kentucky, Mississippi, Tennessee, Arkansas, Louisiana, Oklahoma, Texas; West – Arizona, Colorado, Idaho, New Mexico, Montana, Utah, Nevada, Wyoming, Alaska, California, Hawaii, Oregon, Washington.

Vaccination coverage increased from July to October across most sociodemographic groups (Table 1). The largest increases were found among parents who are ages ≥65 years (prevalence difference [PD] = 15.6, 95%CI: 4.6, 26.6), identify as Hispanic (PD = 8.0, 95%CI: 0.7, 15.3), have higher than college education (PD = 6.2, 95%CI: 2.6, 9.8), have incomes <$35,000 (PD = 13.5, 95%CI: 6.1, 20.9), and have received a COVID-19 vaccination (PD = 6.3, 95%CI: 3.3, 9.3). In adjusted multivariable models, adolescent COVID-19 vaccination coverage was highest among parents who identify as NH Asian (adjusted prevalence ratio [aPR] = 1.11, 95%CI: 1.05, 1.17) or Hispanic (aPR = 1.10, 95%CI: 1.01, 1.21), have an education beyond a college degree (aPR = 1.15, 95%CI: 1.05, 1.25), and had a COVID-19 vaccination (aPR = 5.67, 95%CI: 3.78, 8.51).

Parental intentions to vaccinate their children did not significantly increase from July (69%) to October (71%) (Table 2). Differences in vaccination intent by different sociodemographic characteristics were small; however, the largest differences in intent were found among parents ages ≥65 years (PD = 13.8%, 95%CI: 2.7, 24.9), and households with incomes <$35,000 (PD = 8.9%, 95%CI: 2.1, 15.7). Factors associated with parental intent were being NH Asian (aPR = 1.09, 95%CI: 1.05, 1.14) or Hispanic (aPR = 1.09, 95%CI = 1.01–1.18), and parental COVID-19 vaccination (aPR= 3.89, 95%CI: 2.78, 5.44). Parents of children who were homeschooled were less likely to intend to vaccinate their children (aPR = 0.87, 95%CI: 0.77, 0.99).

**Table 2.** Changes in COVID-19 vaccination intent (vaccinated/probably/definitely) among children 12–17 years by respondent sociodemographic characteristics and survey week, United States, Household Pulse Survey, 21 July 2021 to 11 October 2021.

	7/21–8/2		8/4–8/16		8/18–8/30		9/1–9/13		9/15–9/27		9/29–10/11		Difference		9/29–10/11	
	*n* = 10,154		*n* = 10,762		*n* = 10,750		*n* = 9,816		*n* = 9,170		*n* = 8,459				*n* = 3,798	
	%	(95%CI)	%	(95%CI)	%	(95%CI)	%	(95%CI)	%	(95%CI)	%	(95%CI)	%	(95%CI)	aPR^a^	(95%CI)
**All adolescents (12–17 years)**	69.3	(67.5, 71.1)	69.6	(67.4, 71.7)	70.6	(68.7, 72.5)	73.3	(71.5, 75.2)	71.5	(69.7, 73.2)	70.5	(68.4, 72.6)	1.2	(−1.6, 4.0)		
**Characteristics of Respondent**																
** Age Groups (years)**																
** **18–49	67.9	(65.8, 70.1)	67.2	(64.7, 69.6)	67.6	(65.2, 70.1)	71.0	(68.4, 73.5)	67.7	(65.4, 70.0)	67.7	(64.8, 70.5)	−0.2	(−3.8, 3.4)	1	referent
** **50–64	75.8	(73.3, 78.4)	77.5	(74.2, 80.9)	79.2	(75.8, 82.6)	81.1	(78.5, 83.6)	82.8	(80.4, 85.3)	78.2	(74.1, 82.3)	2.4	(−2.4, 7.2)	1.05	(0.97, 1.12)
** **≥65	60.1	(52.1, 68.2)	69.6	(61.9, 77.3)	73.3	(65.9, 80.7)	69.4	(61.9, 77.0)	69.3	(61.3, 77.2)	73.9	(66.2, 81.5)	13.8	(2.7, 24.9)	0.89	(0.76, 1.04)
** Sex**																
** **Male	71.1	(67.8, 74.4)	70.8	(67.2, 74.4)	72.2	(69.1, 75.4)	75.3	(72.6, 77.9)	71.5	(68.4, 74.6)	71.1	(67.6, 74.7)	0.0	(−4.8, 4.8)	1	referent
** **Female	67.7	(65.9, 69.5)	68.5	(66.4, 70.7)	69.3	(67.2, 71.3)	71.7	(69.6, 73.8)	71.4	(69.5, 73.3)	69.9	(67.6, 72.3)	2.2	(−0.8, 5.2)	0.96	(0.91, 1.02)
** Race/ethnicity**																
** **Non-Hispanic white	65.0	(62.9, 67.0)	63.3	(61.1, 65.4)	64.7	(62.8, 66.7)	68.7	(66.6, 70.8)	66.4	(64.3, 68.5)	67.6	(65.2, 70.0)	2.6	(−0.6, 5.8)	1	referent
** **Non-Hispanic black	69.0	(63.9, 74.1)	66.7	(61.6, 71.8)	71.4	(64.8, 77.9)	74.7	(68.9, 80.5)	75.8	(70.7, 81.0)	66.3	(58.0, 74.5)	−2.7	(−12.4, 7.0)	1.01	(0.89, 1.13)
** **Non-Hispanic Asian	91.0	(87.2, 94.7)	91.5	(88.2, 94.8)	92.6	(88.9, 96.3)	93.6	(89.8, 97.5)	95.9	(93.4, 98.3)	92.1	(88.0, 96.2)	1.1	(−4.5, 6.7)	1.09	(1.05, 1.14)
** **Non-Hispanic other/multiple races	63.1	(55.6, 70.5)	60.5	(49.9, 71.1)	55.7	(47.3, 64.1)	62.3	(53.1, 71.6)	67.9	(60.9, 74.8)	63.7	(56.3, 71.1)	0.6	(−9.9, 11.1)	0.97	(0.83, 1.14)
** **Hispanic	74.6	(70.4, 78.8)	80.1	(76.2, 84.0)	80.7	(77.0, 84.5)	78.8	(74.6, 83.0)	74.3	(69.2, 79.4)	74.2	(69.4, 79.0)	−0.4	(−6.8, 6.0)	1.09	(1.01, 1.18)
** Educational Status**																
** **High school or less	64.4	(60.8, 68.0)	64.0	(60.0, 67.9)	66.2	(62.8, 69.7)	70.0	(66.4, 73.6)	67.9	(64.5, 71.4)	65.6	(62.1, 69.1)	1.2	(−3.8, 6.2)	1	referent
** **Some college or college graduate	70.7	(68.6, 72.9)	72.0	(70.2, 73.7)	71.0	(69.0, 73.0)	73.6	(71.9, 75.2)	71.2	(69.3, 73.0)	71.2	(68.8, 73.7)	0.5	(−2.8, 3.8)	0.97	(0.91, 1.04)
** **Above college graduate	81.7	(79.1, 84.3)	81.0	(78.2, 83.7)	85.3	(83.0, 87.7)	84.7	(82.2, 87.2)	84.0	(81.3, 86.6)	85.1	(82.3, 87.9)	3.4	(−0.4, 7.2)	1.04	(0.96, 1.12)
** Annual Household Income**																
** **Less than $35,000	60.3	(55.8, 64.8)	60.9	(55.5, 66.3)	63.9	(58.9, 68.9)	70.8	(65.9, 75.7)	72.0	(67.5, 76.6)	69.2	(64.1, 74.3)	8.9	(2.1, 15.7)	1	referent
** **$35,000 – $49,999	70.2	(63.6, 76.7)	68.0	(61.0, 75.0)	68.0	(61.7, 74.3)	65.8	(59.1, 72.5)	68.5	(61.5, 75.5)	68.8	(62.2, 75.4)	−1.4	(−10.7, 7.9)	0.90	(0.81, 1.01)
** **$50,000 – $74,999	67.2	(62.1, 72.3)	73.5	(69.3, 77.7)	72.5	(67.9, 77.1)	69.8	(64.7, 74.9)	71.1	(65.5, 76.7)	72.6	(68.2, 76.9)	5.4	(−1.3, 12.1)	0.92	(0.85, 1.00)
** **$75,000 and above	75.8	(73.4, 78.3)	74.9	(72.2, 77.7)	76.1	(73.8, 78.4)	79.8	(77.5, 82.0)	76.2	(73.7, 78.6)	77.3	(74.8, 79.7)	1.5	(−2.0, 5.0)	0.94	(0.87, 1.01)
** **Did not report	68.2	(64.4, 72.1)	67.9	(64.4, 71.3)	68.5	(65.4, 71.6)	71.3	(67.9, 74.8)	67.1	(63.3, 70.8)	64.5	(60.0, 69.0)	−3.7	(−9.6, 2.2)	0.85	(0.77, 0.94)
** Insurance status**																
** **Insured	70.3	(68.2, 72.4)	70.1	(67.7, 72.5)	72.4	(70.5, 74.3)	75.7	(73.6, 77.8)	73.7	(71.9, 75.6)	72.8	(70.7, 74.9)	2.5	(−0.5, 5.5)	1	referent
** **Not insured	63.1	(55.6, 70.7)	69.7	(62.6, 76.9)	62.6	(54.6, 70.6)	63.7	(56.2, 71.2)	71.4	(65.8, 77.0)	69.3	(59.9, 78.7)	6.2	(−5.9, 18.3)	1.11	(0.95, 1.29)
** COVID-19 Status**																
** **Previously diagnosed with COVID-19	63.0	(57.9, 68.0)	62.3	(57.9, 66.7)	63.7	(59.3, 68.1)	67.2	(62.3, 72.2)	64.0	(59.4, 68.6)	62.6	(57.7, 67.4)	−0.4	(−7.4, 6.6)	1	referent
** **Not previously diagnosed with COVID-19	71.2	(69.4, 73.0)	71.6	(69.4, 73.9)	73.0	(71.0, 75.0)	75.8	(73.8, 77.8)	74.0	(72.0, 76.0)	74.0	(71.9, 76.1)	2.8	(0.0, 5.6)	0.97	(0.90, 1.05)
** Respondent COVID-19 vaccination status**																
** **No	20.3	(16.3, 24.2)	21.6	(17.1, 26.1)	22.7	(18.9, 26.4)	24.7	(21.0, 28.4)	19.0	(15.9, 22.2)	17.9	(14.8, 21.0)	−2.4	(−7.4, 2.6)	1	referent
** **Yes	85.1	(83.7, 86.6)	85.1	(83.3, 86.8)	86.0	(84.4, 87.6)	88.0	(86.7, 89.3)	87.6	(86.1, 89.0)	86.8	(85.2, 88.5)	1.7	(−0.5, 3.9)	3.89	(2.78, 5.44)
** Region^b^**																
** **Northeast	75.8	(71.1, 80.6)	75.9	(72.1, 79.7)	76.1	(71.9, 80.2)	80.8	(77.0, 84.6)	76.3	(71.9, 80.7)	73.8	(68.6, 78.9)	−2.0	(−9.0, 5.0)	1	referent
** **Midwest	64.6	(60.1, 69.1)	66.7	(62.5, 71.0)	68.1	(64.6, 71.6)	67.7	(63.6, 71.7)	68.0	(64.9, 71.1)	64.6	(60.3, 68.9)	0.0	(−6.2, 6.2)	1.02	(0.96, 1.08)
** **South	65.3	(62.5, 68.0)	63.5	(60.2, 66.8)	67.5	(65.1, 69.8)	71.4	(68.5, 74.3)	68.8	(65.5, 72.1)	68.9	(65.4, 72.3)	3.6	(−0.8, 8.0)	1.01	(0.94, 1.07)
** **West	75.2	(72.1, 78.3)	77.9	(75.6, 80.2)	74.3	(70.5, 78.2)	75.8	(71.9, 79.8)	75.3	(71.5, 79.2)	75.5	(72.3, 78.6)	0.3	(−4.1, 4.7)	1.02	(0.96, 1.08)
**School type of adolescent (assess for households with only children ages 12–17)**																
** **Only public school	74.8	(71.9, 77.6)	74.0	(71.5, 76.5)	76.2	(73.7, 78.6)	79.5	(77.2, 81.7)	77.5	(74.6, 80.5)	76.3	(73.3, 79.3)	1.5	(−2.6, 5.6)	1	referent
** **Only private school	80.9	(74.2, 87.6)	75.9	(67.2, 84.6)	78.5	(70.6, 86.4)	67.8	(56.8, 78.8)	80.7	(72.8, 88.7)	87.8	(82.9, 92.7)	6.9	(−1.4, 15.2)	1.03	(0.98, 1.09)
** **Only homeschool	66.1	(53.7, 78.5)	54.1	(43.3, 64.9)	51.4	(39.2, 63.6)	66.2	(55.6, 76.7)	44.0	(29.9, 58.1)	60.4	(42.4, 78.4)	−5.7	(−27.6, 16.2)	0.87	(0.77, 0.99)
** **Other combination of schools	71.1	(58.3, 83.8)	65.6	(51.4, 79.8)	74.3	(62.7, 85.9)	75.8	(60.9, 90.7)	60.8	(45.5, 76.0)	72.2	(56.4, 88.0)	1.1	(−19.2, 21.4)	0.98	(0.89, 1.08)
** **None	73.4	(64.3, 82.6)	60.8	(49.2, 72.5)	74.5	(63.6, 85.4)	76.0	(67.3, 84.7)	80.7	(72.3, 89.1)	79.8	(69.2, 90.4)	6.4	(−7.6, 20.4)	1.15	(0.99, 1.34)
** Number of children (12–17) in household (assess for households with only children ages 12–17)**																
** **1	74.8	(72.2, 77.3)	75.1	(72.0, 78.2)	75.9	(73.4, 78.3)	78.5	(75.8, 81.3)	78.9	(76.8, 80.9)	76.3	(73.3, 79.3)	1.5	(−2.4, 5.4)	1	referent
** **2	74.2	(69.9, 78.6)	73.0	(68.7, 77.2)	72.9	(68.4, 77.4)	76.9	(73.1, 80.8)	72.3	(67.0, 77.6)	76.9	(73.6, 80.3)	2.7	(−2.8, 8.2)	1.01	(0.95, 1.06)
** **≥3	69.9	(60.5, 79.2)	57.5	(47.2, 67.7)	65.4	(56.6, 74.3)	63.7	(51.7, 75.6)	64.3	(55.3, 73.3)	67.2	(57.4, 77.0)	−2.7	(−16.2, 10.8)	1.00	(0.90, 1.10)

aPR: adjusted prevalence ratio; CI: confidence interval.

^a^Prevalence ratio adjusted for age, sex, race/ethnicity, educational status, annual household income, insurance status, previous COVID-19 diagnosis, region, school type of adolescent, and number of children in household.

^b^Regions are defined as the following: Northeast – Connecticut, Maine, Massachusetts, New Hampshire, Rhode Island, Vermont, New Jersey, New York, Pennsylvania; Midwest – Indiana, Illinois, Michigan, Ohio, Wisconsin, Iowa, Kansas, Minnesota, Missouri, Nebraska, North Dakota, South Dakota; South – Delaware, District of Columbia, Florida, Georgia, Maryland, North Carolina, South Carolina, Virginia, West Virginia, Alabama, Kentucky, Mississippi, Tennessee, Arkansas, Louisiana, Oklahoma, Texas; West – Arizona, Colorado, Idaho, New Mexico, Montana, Utah, Nevada, Wyoming, Alaska, California, Hawaii, Oregon, Washington.

**Figure 2.**
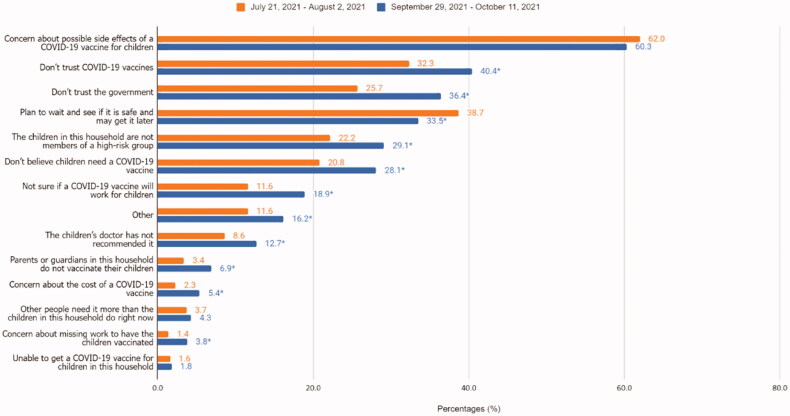
Reasons for not vaccinating adolescent children between 21 July 2021 to 2 August 2021 and 29 September 2021 to 11 October 2021, United States, Household Pulse Survey. *Statistically significant using linear regression to estimate the difference in reasons for not vaccinating between first period of data collection and the latest period of data collection.

Among respondents who did not definitely intend to get their children vaccinated, main reasons for not vaccinating their children in October were concerns about possible side effects (60%), lack of trust in COVID-19 vaccines (40%), lack of trust in the government (36%), and waiting and seeing if it is safe (34%). Reasons for not vaccinating that significantly changed from July to October were lack of trust in the government (from 26 to 36%), lack of trust in vaccines (from 32 to 40%), belief that vaccination is not necessary (from 21 to 28%) or will not work for children (from 12 to 19%), or lack of doctor recommendation (9 to 13%) (Figure 2).

## Conclusion and discussion

Although 61% of adolescents ages 12–17 years have been vaccinated for COVID-19 in October 2021, vaccination coverage only increased by five percentagepoints between July and October, and parental intention to vaccinate their adolescent children appear to have plateaued. While larger increases in vaccination uptake were observed among older parents, those who identify as Hispanic, those with higher levels of education, and those with annual household incomes <$35,000, many groups still had low vaccination uptake with no changes throughout the summer and early fall of 2021. The lack of change in percentage of parents who intend to vaccinate their adolescent children suggest that COVID-19 adolescent coverage will continue in its current trajectory unless tailored messages and interventions are made to increase vaccination uptake in this population. In addition, almost one fifth of parents were reluctant to vaccinate their adolescent children – a trend that has been consistent since July. This is similar to findings from other polls that have found that 21% of parents do not definitely plan to vaccinate their adolescent children [4]. Data from the CDC also found a lag in national adolescent vaccination estimates throughout the summer and early fall of 2021 [14]. Low vaccination coverage can increase risk for severe illness from COVID-19, particularly for children who have obesity, diabetes, asthma or chronic lung disease, sickle cell disease, or immunosuppression [15]. It is important to achieve high vaccination coverage to prevent against risk of severe infection, safely resume in-person learning in schools, sports, and other activities, as well as to prevent the transmission of the virus.

Parental COVID-19 vaccination status was strongly associated with adolescent vaccination coverage and parental intent to vaccinate their adolescent children. Parents who were vaccinated for COVID-19 were significantly more likely to vaccinate or intend to vaccinate their adolescent children for COVID-19, underscoring the urgency of discussing with parents about the importance of vaccination not only for themselves but also for their children. Barriers to vaccination uptake among adults may also be similar to barriers to vaccination uptake for children. Previous studies have found similar disparities in vaccination coverage and reasons for not vaccinating among adults [16,17]. For example, previous studies found that adults who identified as non-Hispanic Black or non-Hispanic other/multiple races were less likely to be vaccinated or intend to be vaccinated against COVID-19, with concerns about side effects as the main reasons for non-vaccination. Identifying barriers to uptake and addressing parental concerns about vaccination for themselves may help boost confidence in vaccines and vaccinations for their children [18–21].

While the percentage of adults who are unsure or reluctant to vaccinate their adolescent children have not changed very much since July, their reasons for not vaccinating have changed. Although the main reason for parents not vaccinating their children continues to be concern about side efforts, other reasons such as lack of trust in the government and in the vaccine, as well as the beliefs that the vaccine is not needed or effective have increased in October compared to July. This suggests the need for increased efforts to build trust, provide clear messages about vaccine safety and efficacy, and encourage providers to recommend the vaccine are needed. Increasing trust in vaccines, providers, and governments require increased transparency in communications about the process for authorising, approving, making recommendations for, and monitoring the safety of COVID-19 vaccines, as well as addressing and stopping the spread of vaccine misinformation [22].

The findings in this study are subject to several limitations. First, although sampling methods and data weighting were designed to produce nationally representative results, respondents might not be fully representative of the general U.S. adult population. Second, vaccination status for respondents and their adolescent children was self-reported by respondents and may be subject to social desirability bias. Third, the survey was only able to collect information from respondents and not their adolescent children; as a result, vaccination intentions among adolescents are unknown, which may differ from parental intentions. Fourth, the survey did not collect information on vaccination coverage and intent of children ages 5–11 years, for which the COVID-19 vaccines were recently approved [23]. Finally, the HPS has a low response rate (<10%); although non-response bias assessment conducted by the Census Bureau found that the survey weights mitigated most of this bias [24].

Since the vaccine is now approved for children ages five years and older, it is important for all age-eligible children to be fully vaccinated for COVID-19. As children and adolescents resume in-person learning at school and engage in other social activities, having high and equitable vaccination coverage is needed to prevent serious health outcomes, particularly for children who have co-morbidities. Given that provider recommendation is often the most potent predictor of parental vaccine decisions [25–27], healthcare providers should offer COVID-19 vaccines at every visit, address any parental concerns, and highlight the importance of being fully vaccinated and the safety and efficacy of vaccines. A higher percentage of parents reported that not receiving a provider recommendation was a reason for not vaccinating their child in October compared to July, signifying the impact of provider recommendations on vaccination coverage in this population. In addition to receiving more information about vaccine safety and efficacy, other studies have found that having a school requirement would increase parents’ intentions to vaccinate their adolescent children [28–30]. While most K-12 schools have not issued requirements for COVID-19 vaccination [31], promoting vaccination can help schools safely return to in-person learning, sports, and extracurricular activities [18].

As vaccine distribution moves to primary care settings [32], and more children are eligible for the vaccine, healthcare providers are in a greater position to make a difference in childhood vaccination by having conversations with their patients, addressing misinformation and concerns, and providing tailored vaccine information to patients. Studies also found that most adolescents receive vaccinations at their usual doctor’s office or clinic [32,33], underscoring the critical role of healthcare providers in increasing vaccination uptake and confidence among this population. Boosting confidence and trust in vaccine safety and efficacy is essential in protecting children and families from COVID-19 and safely resuming pre-pandemic activities. 

## Note

1 Region is categorised as Northeast (Connecticut, Maine, Massachusetts, New Hampshire, Rhode Island, Vermont, New Jersey, New York, Pennsylvania), Midwest (Indiana, Illinois, Michigan, Ohio, Wisconsin, Iowa, Nebraska, Kansas, North Dakota, Minnesota, South Dakota, Missouri), South (Delaware, District of Columbia, Florida, Georgia, Maryland, North Carolina, South Carolina, Virginia, West Virginia, Alabama, Kentucky, Mississippi, Tennessee, Arkansas, Louisiana, Oklahoma, Texas); and West (Arizona, Colorado, Idaho, New Mexico, Montana, Utah, Nevada, Wyoming, Alaska, California, Hawaii, Oregon, Washington).
